# Early Adopters of Apple Health Records at a Large Academic Medical Center: Cross-sectional Survey of Users

**DOI:** 10.2196/29367

**Published:** 2022-01-25

**Authors:** Joshua Rolnick, Robin Ward, Gordon Tait, Neha Patel

**Affiliations:** 1 United States Department of Veterans Affairs Philadelphia, PA United States; 2 Perelman School of Medicine University of Pennsylvania Philadelphia, PA United States; 3 University of Pennsylvania Health System Philadelphia, PA United States

**Keywords:** Apple Health Records, personal health record, electronic health record, patient satisfaction, early adopters, cross-sectional survey

## Abstract

**Background:**

Mobile applications offer a new approach to personal health records, which are internet-based tools for patients to consolidate and manage their health information. The University of Pennsylvania Health System (UPHS) was one of the first health systems to participate in Apple Health Records (AHR), a prominent example of this new generation of personal health records.

**Objective:**

This study aimed to characterize early adoption of AHR among UPHS patients and understand user perspectives.

**Methods:**

An email-based survey with fixed answer, Likert scale, and open-ended questions was administered to all UPHS patients using AHR in the first 10 months of enrollment. Survey data linked to the UPHS electronic health record system were used to analyze responses. Multivariable logistic regression modeled the association of patient characteristics with user ratings. Content analysis was used to analyze open-ended questions.

**Results:**

At the time of the survey, a total of 1458 patients had used AHR at least once. Mean age of AHR users was 47.5 years, 66.3% (967/1458) were male, 70.9% (1033/1458) were white, and 80.8% (1178/1458) had private insurance. Response rate was 26.8% (391/1458); 46.3% (180/389) were very satisfied with AHR, and 67.7% (264/390) described it as very easy to use. The most commonly utilized features were lab results (324/391, 82.9%), clinical vitals (264/391, 67.5%), and medications (253/391, 64.7%). No patient characteristics were associated with reporting high satisfaction or ease of use. The most common reason for using AHR was convenience/ease of use, and 58.2% (160/275) of users reported allowing no other apps to access their health information, citing privacy as one consideration.

**Conclusions:**

Early adopters of AHR were demographically white, male, and privately insured. Convenience was an important facilitator, and users were selective in which apps they allowed to access their health information.

## Introduction

Despite near universal adoption of electronic health records (EHRs) in the United States, interoperability and data fragmentation remain significant challenges [1]. One proposed solution is the “personal electronic health record,” an internet-based set of tools that allow people to directly consolidate, access, and control their health information [1,2]. Development of a successful personal health record has been a policy objective since the creation of the National Coordinator for Health Information Technology in the Bush Administration [3].

Early attempts to develop personal health records by companies such as Google and Microsoft failed to achieve widespread adoption [3]. Lack of perceived usefulness, low trust, and a lack of meaningful integration of hospital data were among the barriers limiting use [4]. In the last few years, a newer generation of mobile application–based personal health records has been developed. One example is Apple Health Records (AHR), a feature within the Apple Health app available on Apple iPhones that incorporates health care data from multiple health care sources and allows users to see integrated data simultaneously. In 2018, Apple partnered with 12 large health systems, including the University of Pennsylvania Health System (UPHS), to provide their patients with AHR. AHR has since expanded to more than 500 health systems [5]. AHR has significant potential for usage since iPhones are used by more than half the US population [6].

Yet, despite the number of health systems participating in the AHR pilot, little is known about the characteristics of AHR users and their experiences. To address this gap, we analyzed UPHS user characteristics and administered a survey to UPHS patients who had enabled the AHR feature.

## Methods

### Study Design

This study utilized a cross-sectional survey of AHR users at a large academic medical center. Survey results were linked to EHR data on demographics and health care utilization. Content analysis of open-response questions was performed.

AHR allows users to aggregate information from multiple health systems into a single interface within the iPhone health app. It gathers different forms of health information, including medications, vital signs, lab results, and procedures, integrating information from multiple sources into a single category for each information type. AHR thereby permits users to access organized information pooled from individual EHR systems on a single personal device. Users receive notifications when their health record is updated by a participating health system. Since this survey was administered, AHR has added features to perform longitudinal analysis of pooled data and share results with health care providers [7].

### Human Subjects Research Review

This study was deemed excluded from human subjects review as a quality improvement project by the University of Pennsylvania institutional review board.

### Participants

UPHS launched its pilot of AHR in January 2018. AHR is accessible through the Health application on an iPhone device. UPHS patients can add their UPHS data to the app using their UPHS patient portal credentials for authentication. Patients without an active portal account were directed to a website to create one. Participants included all UPHS users of AHR in the first 10 months of the pilot, defined as anyone who enabled AHR to download information to their mobile device.

### Survey Instrument and Recruitment

A survey was developed by the study authors (see Multimedia Appendix 1). The survey was not based on a specific prior survey instrument but was developed to fit the intended purpose of capturing information from AHR users. The survey was designed using principles of survey design for Likert scale and open-response surveys and utilized standard categories for Likert scales. In addition, the survey was iteratively refined, initially through feedback from UPHS staff members, followed by pilot administration to a subsample of AHR users in September 2018. The final survey included 11 Likert scale, multiple selection, and open-ended questions. The survey ran from September 2018 to November 2018 and was administered using the Alchemer survey platform. Eloqua was used for email outreach and tracking. Users were sent an initial email with the survey link and up to 2 follow-up emails for nonresponse.

### Demographic and Utilization Characteristics

Information was extracted on user age, sex, race, primary insurance, and health system utilization from the UPHS EHR system. Data were linked to survey responses, and the linked data were deidentified.

### Analysis

Demographic and utilization characteristics were summarized for all users, and survey respondents were compared with nonrespondents. Differences between respondents and nonrespondents were compared using *t* tests for normal continuous variables, Wilcoxon rank-sum tests for nonnormal continuous variables, and chi-square tests for categorical variables. Patient portal users were compared with AHR users by identifying standardized differences greater than 0.2 [8]. This approach was used because the large sample size rendered even small differences statistically significant. Multivariable logistic regression was used to analyze the association of respondent characteristics with self-reported high satisfaction and ease of use. This analysis was considered exploratory, and model covariates were chosen based on demographic and utilization variables used to describe AHR and patient portal users. A multivariable analysis was performed in which age, hospitalizations, office visits, and active medications were included as continuous variables, sex was included as a binary variable, and race and primary insurance were included as categorical variables with the categories listed in Table 1. A *P* value <.05 was considered statistically significant. This threshold was set prior to all analyses. Satisfaction was dichotomized as very satisfied or not, and ease of use was categorized as reporting that AHR was very easy to use or not.

Open-ended responses were coded using content analysis. Two study investigators (JR and NP) separately coded all responses. Discrepancies were reviewed and resolved by mutual agreement.

Stata 14.2 was used for all data analyses.

**Table 1:** Demographic and utilization characteristics of users of Apple Health Records (AHR).

Characteristics			All patient portal users (N=535,422), n (%)		All AHR users (n=1458), n (%)		Survey responders^a^ (n=373), n (%)		Survey nonresponders (n=1085), n (%)		*P* value^b^ (n=1085)
Age (years), mean (SD)			50.0 (17.2)		47.5 (14.9)		50.0 (15.1)		46.6 (14.8)		<.001
Male, n (%)			195,223 (36.5)		967 (66.3)		289 (77.5)		678 (62.5)		<.001
**Race, n (%)**											
	White	382,552 (71.4)		1033 (70.9)		296 (79.4)		737 (67.9)		<.001	
	Black	87,567 (16.4)		210 (14.4)		35 (9.4)		175 (16.1)			
	Asian	22,915 (4.3)		90 (6.2)		16 (4.3)		74 (6.8)			
	Other	21,791 (4.1)		78 (5.3)		19 (5.1)		59 (5.4)			
	Unknown	20,597 (3.8)		47 (3.2)		7 (1.9)		40 (3.7)			
**Primary insurance, n (%)**											
	Medicare	49,946 (9.3)		212 (14.5)		79 (21.2)		133 (12.3)		<.001	
	Medicaid	25,958 (4.9)		47 (3.2)		13 (3.5)		34 (3.1)			
	None	7919 (1.5)		21 (1.4)		1 (0.3)		20 (1.8)			
	Private	451,599 (84.3)		1178 (80.8)		280 (75.1)		898 (82.8)			
Hospitalizations^c^, median (IQR)			0 (0-1)		0 (0-2)		0 (0-2)		0 (0-2)		.053
Office visits^c^, median (IQR)			1 (0-3)		2 (1-5)		2 (1-5)		2 (1-5)		.63
Active prescriptions^c^, median (IQR)			2 (0-6)		4 (1-10)		4 (0-11)		4 (1-10)		.82

^a^The number of respondents differs in Tables 1 and 2 because there were 18 survey responses with email addresses that could not be matched to a unique University of Pennsylvania Health System (UPHS) electronic health record. Reasons for nonmatching included patients who changed their UPHS email address and email addresses shared between multiple AHR users (eg, in the same household).

^b^Respondents compared with nonrespondents.

^c^Over 12 months prior to survey administration.

## Results

### Survey Responses and Respondent Characteristics

There was a total of 1458 UPHS AHR users at the time of survey administration. Mean user age was 47.5 (SD 14.9) years, 66.3% (967/1458) were male, and 70.9% (1033/1458) were White (Table 1). Median number of hospitalizations was 0 (IQR 0-2). Of 1458 users, 68.8% (1003/1458) opened the email (unique open rate), 29.1% (424/1458) accessed the survey (click-through rate), and 26.8% (391/1458) submitted a response. Survey respondents were older, more likely to be male, more likely to be White, and more likely to have private insurance than survey nonrespondents. There were no statistically significant differences in hospitalizations, office visits, or prescriptions. Although AHR users were majority male, in contrast, only a minority of patient portal users were male (195,223/535,422, 36.5%; standardized difference 0.63). In addition, AHR users had higher utilization than all patient portal users over 12 months, including more hospitalizations (standardized difference 0.21), more office visits (standardized difference 0.25), and more active prescriptions (standardized difference 0.21).

Of the 391 participants, 180 (46.3%) were very satisfied with AHR, whereas 10 (2.6%) were very dissatisfied (Table 2). Although 264 (67.7%) of the 391 participants described AHR as very easy to use, 6 (1.5%) called it very difficult. On a 1 (low) to 9 (high) scale, respondents reported a mean 7.7 (1.9) likelihood to recommend to a friend. The most commonly used features were lab results (324/391, 82.9%), clinical vitals (264/391, 67.5%), and medications (253/391, 64.7%). Respondents most often shared information with family (191/391, 48.8%), followed by not sharing with anyone (138/391, 35.3%) and sharing with a physician (137/391, 35.0%). The most frequent means of finding out about AHR was through news media (142/391, 36.3%).

**Table 2:** User survey responses.

Response			Results
**Overall satisfaction, n (%)**			
	Very satisfied	180 (46.3)	
	Satisfied	152 (39.1)	
	Neutral	39 (10.0)	
	Dissatisfied	8 (2.1)	
	Very dissatisfied	10 (2.6)	
**How easy to use, n (%)**			
	Very easy	264 (67.7)	
	Somewhat easy	99 (25.4)	
	Neutral	18 (4.6)	
	Difficult	3 (0.8)	
	Very difficult	6 (1.5)	
How likely to recommend to a friend (1-9), mean (SD)			7.7 (1.9)
**Features used, n (%)**			
	Lab results	324 (82.9)	
	Clinical vitals	264 (67.5)	
	Medications	253 (64.7)	
	Conditions	183 (46.8)	
	Procedures	173 (44.2)	
	Immunizations	160 (40.9)	
	Allergies	141 (36.1)	
	All records	14 (3.6)	
	Other	13 (3.3)	
**Discussed information in AHR^a^, n (%)**			
	Family	191 (48.8)	
	Did not share with anyone^b^	138 (35.3)	
	Physician	137 (35.0)	
	Friend	76 (19.4)	
	Other members of care team	56 (14.3)	
	Pharmacists	19 (4.9)	
	Other person	6 (1.5)	
**How found out about AHR, n (%)**			
	News article	142 (36.3)	
	Email announcement	75 (19.2)	
	Friend	20 (5.1)	
	Family	14 (3.6)	
	Physician	10 (2.6)	
	Other member of care team	6 (1.5)	

^a^AHR: Apple Health Records.

^b^Did not share results (ie, no one).

In adjusted models, no demographic or utilization characteristics were significantly associated with reported satisfaction with AHR or ease of use (Table 3).

**Table 3:** Association of user characteristics with ratings of Apple Health Records.

Characteristics	Very satisfied, OR^a^ (95% CI)	*P* value	Very easy to use, OR (95% CI)	*P* value
Age	1.00 (0.99-1.02)	.79	1.00 (0.98-1.02)	.83
Male	0.89 (0.53-1.49)	.65	0.83 (0.47-1.46)	.52
Race	1.05 (0.84-1.31)	.69	1.03 (0.81-1.32)	.80
Primary insurance	1.13 (0.87-1.46)	.37	1.19 (0.91-1.56)	.19
Hospitalizations	0.87 (0.65-1.15)	.32	0.88 (0.65-1.19)	.40
Office visits	1.00 (0.94-1.08)	.90	0.99 (0.92-1.07)	.79
Active prescriptions	1.00 (0.99-1.02)	.66	1.01 (0.99-1.02)	.45

^a^OR: odds ratio.

### Responses to Open-Ended Questions

The most common reason that respondents gave for using AHR was convenience/ease of use (138/305, 45.2%), followed by having all information in one place (54/305, 17.7%; Table 4). One respondent noted that “[i]t was very easy and I don't have to log in to view them.” Asked what other information they wanted to be included, respondents indicated, at near equal rates, no other information and all medical information. For example, one respondent said “[i]'d like to have EVERYTHING that Penn Medicine has in my patient record accessible through Apple Health Records.” The most frequent request for change to AHR was to provide better labeling and displays.

When asked what other apps respondents allowed to access their health information, the most common answer (160/275, 58.2%) was “none.” Privacy was cited as one reason. One respondent explained: “As you know, privacy is a major concern with health records. Apple has a reputation for being very serious about customer privacy. I would never grant access to health records to companies like Google or Facebook, whose business model rests on selling privacy.”

**Table 4:** Coded responses to open-response questions.

Questions and responses			Responses, n (%)^a^		Example quotations
**Approximately how much time have you spent using Apple Health Records?^b^ (n=222)**					
	Not much	32 (14.4)		-^c^	
	<1 hour	134 (60.4)		“About a half-hour or so reviewing records.”	
	1 to 2 hours	28 (12.6)		-	
	2 to 3 hours	10 (4.5)		-	
	≥3 hours	18 (8.1)		-	
**Why did you choose to access your Penn health information via Apple Health Records? (n=305)**					
	Convenience/ease of use	138 (45.2)		“It was very easy and I don't have to log in to view them”	
	Information in one place	54 (17.7)		“I love having all of my information in one place. I don't want to have an app for each provider. I am confident that Apple is keeping measures to keep my information secure”	
	Experimental/curiosity	47 (15.4)		-	
	Apple production	42 (13.8)		-	
	Portability	13 (4.3)		-	
	Access specific information	11 (3.6)		-	
**What other health information would you like to have through your Apple Health Records? (n=181)**					
	No other information	50 (27.6)		-	
	Everything	49 (27.1)		“I'd like to have EVERYTHING that Penn Medicine has in my patient record accessible through Apple Health Records.”	
	Appointments	26 (14.4)		-	
	Radiology	26 (14.4)		-	
	Clinical notes	13 (7.2)		-	
	Additional lab data	10 (5.5)		-	
	Communication with care team	7 (3.9)		-	
**If you could change one thing about Apple Health Records, what would it be? (n=170)**					
	No changes	63 (37.1)		-	
	Better display, labeling, and analysis	44 (25.9)		“Better visualization of test results, etc. changing over time. Graphs would be good.”	
	Add providers and health systems	23 (13.5)		-	
	Access to actual reports, imaging, and documentation	17 (10)		-	
	Integration with information from other sources	16 (9.4)		-	
	Change units	7 (4.1)		-	
**What other apps have you allowed to access your health record data? (n=275)**					
	None	160 (58.2)		“None. As you know, privacy is a major concern with health records. Apple has a reputation for being very serious about customer privacy. I would never grant access to health records to companies like Google or Facebook, whose business model rests on selling privacy.”	
	Fitness trackers	69 (25.1)		-	
	Patient portal	23 (8.4)		-	
	Other health systems patient portals	13 (4.7)		-	
	Other Apple products	10 (3.6)		-	

^a^Totals differ by question due to nonresponse, and numbers for each question with codable responses are shown.

^b^Time period refers to overall usage as an Apple Health Record user.

^c^Example not listed.

## Discussion

### Principal Findings

In this survey of patients using AHR at an early health system adopter, most respondents reported high satisfaction and ease of use, and none of the tested characteristics was associated with differences in ratings. Convenience/ease of use was a frequently cited rationale for use, and many respondents requested incorporation of all health information into AHR. This study has several notable findings for future use of mobile application–based personal health records such as AHR.

First, personal health records may be used by specific patient demographics. Most patients were White and privately insured. In comparison with all patient portal users, AHR users were disproportionately male. Disparities are a concern with mobile application–based personal health records, because smartphone users are younger and wealthier than the US population [9,10]. Older patients have lower digital health literacy than younger patients [11], although such disparities are decreasing over time [12]. Yet, demographics also were not associated with differences in AHR ratings. In addition, although AHR users had higher health care utilization than patient portal users, utilization remained low, with a low number of office visits and predominantly no hospitalizations in the year prior to the study, suggesting that the app was not reaching patients with high health system utilization who may have the most significant need to share information because of frequent hospitalizations or complex care. The gender disparities in use are surprising given the female-predominant overall patient portal population and require further investigation to understand how barriers and facilitators of use disproportionally affect female patients.

Second, convenience was an important facilitator of use, and most AHR users were selective about which apps accessed their health information. For personal health records to achieve widespread use, they will need to overcome several barriers. Platforms will need to be convenient and provide the privacy protections for patients to feel comfortable with storage of substantial health information.

Indeed, privacy considerations and trust in the Apple brand to safeguard information emerged as important factors, a finding consistent with past studies of personal health records [13]. Safeguarding of personal information will be crucial to personal health record success, as security lapses leading to stories of compromised personal information may raise privacy concerns among would-be users.

### Comparison With Prior Work

These findings advance our understanding of personal health records. Prior work has examined utilization and factors affecting adoption of national personal health records in countries such as England and Portugal [14,15]. Other work sought to understand the barriers to adoption of earlier personal health records such as Google Health [4]. In addition, commentators have noted the potential advantages of mobile application–based personal health records offered by private vendors, including AHR [1,16]. This study offers new insight into the characteristics and perspectives of AHR users at one of the first health systems to participate in the AHR pilot.

Empowering patients with their health records has been a goal for the Office of the National Coordinator (ONC) for Health Information Technology. In 2016, Congress passed the 21st Century Cures Act to drive electronic access, exchange, and use of health information. The ONC Cures Act Final Rule implemented the interoperability provisions of the Cures Act to promote patient control over their own health information [17].

The rule requires the health care industry to adopt standardized application programming interfaces (APIs) to allow patients to securely access structured electronic health information using smartphone applications. These changes will make it easier for third-party apps to access and integrate health data, including AHR, which uses these same standards for data exchange. AHR is utilizing these standards to add new data-sharing features, including bidirectional sharing that will allow users to directly share AHR data with certain AHR systems [18]. Understanding the barriers and facilitators of using these tools is crucial to guide future development in a direction that will meet the needs of patients and potentially address health disparities that could result from wider adoption. It will also be necessary for information policy development in this area.

### Limitations

This study has several limitations. It reported results from a single-center experience with one platform. Results may not generalize to other health systems or platforms. That said, this early experience with AHR adds to our knowledge of an important new exemplar of personal health records. This study also found some differences between respondents and nonrespondents, and respondents may not be representative of the overall user base. Further efforts will be needed with larger-scale surveys and qualitative studies. Third, this study provided only information from self-reports. Finally, this study examined only patient perspectives and not how AHR use might affect clinical outcomes.

In summary, we reported characteristics and perspectives of patient users at an early adoption of AHR. Our experiences offer several lessons for future use and study of personal health records.

## Appendix

Multimedia Appendix 1 Supplemental survey.

## Abbreviations

AHR: Apple Health Records
API: application programming interface
EHR: electronic health record
ONC: Office of the National Coordinator
UPHS: University of Pennsylvania Health System
