# Supplementary material for: Early Adopters of Apple Health Records at a Large Academic Medical Center: Cross-sectional Survey of Users
Source: J Med Internet Res. 2022 Jan 25;24(1):e29367. doi: 10.2196/29367 (PMC8826150; doi:10.2196/29367)
Supplement: Multimedia Appendix 1 [file jmir_v24i1e29367_app1.docx]

Multimedia Appendix 1. Survey.

**/INTRODUCTION/**

Penn Medicine would like feedback from its patients on Apple’s new Health Records Feature. With this new feature you access your Penn Medicine health record from the Health app on your iPhone at any time. An example of how you view this new feature is below.

[Example]

1. Have you used the new Apple Health Records feature?

Yes/No/Unsure

**/IF YES/**

1. Overall, how satisfied are you with Apple Health Records?

very dissatisfied (1), somewhat dissatisfied, nether satisfied nor dissatisfied, somewhat satisfied, very satisfied (5)

1. How easy was it to use Apple Health Records?

Very difficult (1), somewhat difficult, neither difficult nor easy, somewhat easy, very easy (5)

1. Which of the following have you used within your Apple Health Records?

**/CHECKBOX/** All Records, Allergies, Clinical Vitals, Conditions, Immunizations, Lab results, Medications, and Procedure

1. With whom have you discussed the information found in your Apple Health Records?

**/CHECKBOX/** Friend(s), Family, Doctors, Other members of my care team (nurse, or nurse practitioner, or physician assistant), Pharmacist, No One, or Other **/FREE TEXT/**

1. Approximately how much time have you spent using Apple Health Records? **/FREE TEXT/**
2. How did you find out about Apple Health Records?

**/CHECKBOX/** Friend(s), Family, Doctor, Email, News Article, Other **/FREE TEXT/**

1. Why did you choose to access your Penn health information via Apple Health Records? **/FREE TEXT/**
2. What other health information would you like to have through your Apple Health Records? **/FREE TEXT/**
3. If you could change one-thing about Apple Health Records, what would it be? **/FREE TEXT/**
4. How likely are you to recommend Apple Health Records to your friends and/or family?

Scale 1(not likely)-9(very likely)

1. What other apps have you allowed to access your health record data? **/FREE TEXT/**
